# Undetectable proviral deoxyribonucleic acid in an adolescent perinatally infected with human immunodeficiency virus-1C and on long-term antiretroviral therapy resulted in viral rebound following antiretroviral therapy termination: A case report with implications for clinical care

**DOI:** 10.1097/MD.0000000000018014

**Published:** 2019-11-22

**Authors:** Catherine Kegakilwe Koofhethile, Sikhulile Moyo, Kenanao Peggy Kotokwe, Patrick Mokgethi, Lorato Muchoba, Selebogo Mokgweetsi, Tendani Gaolathe, Joseph Makhema, Roger Shapiro, Shahin Lockman, Phyllis Kanki, M. Essex, Simani Gaseitsiwe, Tulio de Oliveira, Vladimir Novitsky

**Affiliations:** a Botswana Harvard AIDS Institute Partnership, Gaborone, Botswana; b Harvard T.H. Chan School of Public Health, Boston, MA; c College of Health Sciences, Nelson R Mandela School of Medicine, University of KwaZulu-Natal (UKZN); d KwaZulu-Natal Research Innovation and Sequencing Platform (KRISP), UKZN, Durban, South Africa.

**Keywords:** adolescent, Botswana, HAART, HIV, viral rebound

## Abstract

**Rationale:**

Early initiation of antiretroviral therapy (ART) leads to long-term viral suppression, reduces proviral reservoir size, and prolongs time to rebound. Since human immunodeficiency virus (HIV) is a lifelong disease, diagnostic monitoring after confirmed infection is typically not performed; therefore, little is known about the impact of early initiation and long-term ART on the sensitivity of assays that detect HIV antibodies and viral nucleic acid in children and adolescents.

**Patient concerns:**

Here we report 1 case of diagnosed and confirmed perinatal HIV-1C infection with longstanding viral suppression, who subsequently had a negative HIV-1 deoxyribonucleic acid (DNA) test, undetectable antibodies to HIV-1, and high CD4+ T cell count after 14 years of ART.

**Diagnosis:**

The patient was diagnosed with HIV in 2002 at 1 and 2 months of age using DNA polymerase chain reaction. At 8 months old, his viral load was 1210 HIV ribonucleic acid (RNA) copies/mL and CD4 T cell count was 3768 cells/mm^3^.

**Intervention:**

At the age of 9 months, highly active antiretroviral therapy comprising of zidovudine, nevirapine, and lamivudine was initiated. The patient remained on this treatment for 14 years 11 months and was virally suppressed.

**Outcomes:**

At the age of 14 years 4 months, the participant decided to visit a local voluntary HIV testing center, where a rapid HIV test came out negative and the viral load was undetectable (<400 HIV-1 RNA copies/mL). These results led to termination of ART which led to viral rebound within 9 months.

**Lessons:**

As more people with early HIV infection initiate early ART in the context of “Test and Treat all” recommendations, aspects of this report may become more commonplace, with both clinical and public health implications. If the possibility of functional cure (or false-positive diagnosis) is being considered, decisions to terminate ART should be made cautiously and with expert guidance, and may benefit from highly sensitive quantification of the proviral reservoir.

## 1 Introduction

There is currently no cure or an effective vaccine against human immunodeficiency virus (HIV). Antiretroviral therapy (ART) can result in full suppression of HIV replication but does not eliminate the virus due to existence of proviral reservoir. The proviral reservoir, comprised of a pool of latently infected cells, is a major obstacle to achieving a cure. During the early phase of HIV infection, proviral deoxyribonucleic acid (DNA) is harbored in multiple cells such as long-lived CD4+ T cells in the periphery and sanctuaries, establishing latency.^[1–4]^ Viral replication and rebound can occur following reactivation of these latently infected cells, particularly in the absence of ART.^[5–9]^ Hence, HIV-infected individuals should remain on treatment for life. With the current “Test and Treat all” recommendations^[10]^ caution must be exercised in clinical management of cases with long-term ART and viral suppression.

Currently, early ART initiation among infants perinatally infected with HIV is common, resulting in many HIV-positive children and adolescents receiving long-term ART. Botswana and other countries demonstrated in clinical trials that ART can prevent most (>98%) mother-to-child HIV transmissions,^[11–17]^ and that early initiation of ART in the breakthrough cases is feasible.^[18–20]^ Early infant diagnosis and initiation of ART has also been shown to be of critical importance in reducing infant morbidity and mortality.^[21,22]^

Recent studies on proviral HIV reservoirs suggest that early initiation of ART reduces the size of the reservoir and prolongs time to virus rebound, while ART interruption leads to virus rebound.^[4,23]^ What remains unknown is the impact of combining early ART initiation and long-term ART on the size of the proviral reservoir and detection of HIV antibodies and viral nucleic acid. In virologically suppressed HIV-infected individuals, rapid tests for HIV-1 antibody and/or nucleic acid tests could produce false-negative results, which may mislead health care providers and patients. Here we report a case from a routine clinical practice in Botswana in which a perinatally HIV-infected adolescent tested negative for HIV antibodies after 14 years of ART, stopped their ART and experienced viral rebound 9 months later.

## 2 Case history

We present a case study of an adolescent male (“the participant”) with confirmed perinatal HIV infection within 1 month of life. Accompanying records (Table 1) indicated that the participant's mother was enrolled in 2002 in a prevention of mother-to-child transmission (PMTCT) clinical trial known as “The Mashi study” (ClinicalTrials.gov Identifier: NCT00197587; 2002–2005) with no prior ART history and was given standard prophylaxis of nevirapine (NVP) and zidovudine (AZT) from 34 weeks’ gestation through delivery.^[18,19]^ The mother was randomized to the breastfeeding arm of the study. The mother had a viral load of 2,090 HIV-1 ribonucleic acid (RNA) copies/mL at the time of delivery.

**Table 1:**
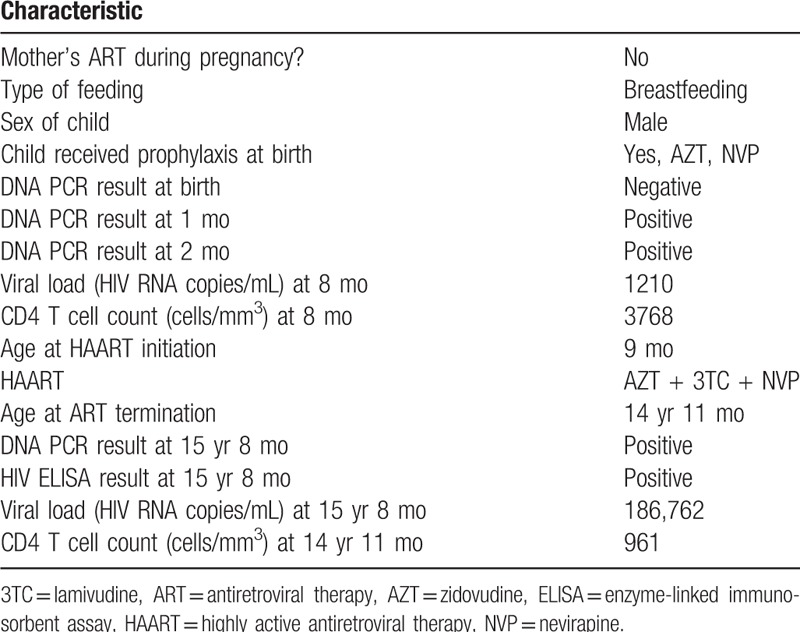
Diagnostic and clinical events of the case.

**Figure 1:**
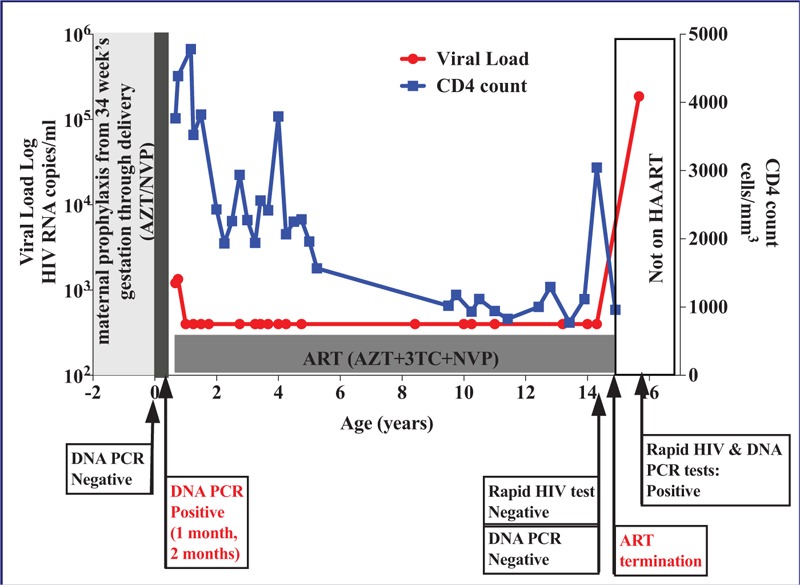
Longitudinal clinical and laboratory data for case 097-533.

HIV DNA polymerase chain reaction (PCR) for the baby at birth (negative), 1 month (positive), and 2 months (positive) after birth was performed using the Roche Amplicor, version 1.5 (Roche Molecular Systems, Inc, Branchburg, NJ). The participant initiated ART (AZT + lamivudine + NVP) at 9 months of age, and remained on this regimen for 14 years (Fig. 1). The participant had high median follow-up CD4+ T cell count of 2068 cells/mm^3^ (interquartile range 1117–2938) and consistent viral suppression (<400 copies/mL) over the 14 years of routine follow-up in a local clinic.

At the age of 14 years 4 months, the participant decided to visit a local voluntary HIV testing center, where a rapid HIV test was negative and the viral load was undetectable (<400 HIV-1 RNA copies/mL). To confirm these results, additional HIV rapid tests – determine HIV-1/2 (Abbott Laboratories, North Chicago, IL) and UniGold HIV (Trinity Biotech, Ireland) - and a DNA PCR test - COBAS AmpliPrep/COBAS TaqMan (CAP/CTM) HIV-1 Qualitative Test (Roche Molecular Systems, Inc), were performed, all with negative results. The test results were discussed extensively among the participant, his parent and health care provider. The decision was made to terminate ART when the participant was aged 14 years 11 months. The authors of this report did not play any role in the decision to terminate ART.

Using the Mashi study records, the participant at the age of 15 years 8 months was approached and recruited to the new HIV reservoir study in 2018. The study aimed at assessing the serological, virological outcomes, and proviral reservoir in adolescents during long-term ART. The study included adolescents (15–17 years of age) perinatally infected with HIV who received ART for >10 years.

The viral load at enrollment (9 months after ART termination) was 186,762 HIV RNA copies/mL. Two HIV enzyme-linked immunosorbent assay tests – Murex HIV-1.2.O (Murex Biotech, Dartford, UK) and Biorad Genetic Systems HIV-1/HIV-2 Plus O (Redmond, WA) – and the HIV DNA PCR test – CAP/CTM HIV-1 qualitative test – were also positive at enrollment in the HIV reservoir study. The test results demonstrate that despite negative HIV rapid and DNA PCR test results at age 14, the participant had latent infection, and termination of ART led to viral rebound.

## 3 Discussion

The case report describes an adolescent male who received HIV prophylaxis (AZT, NVP) and tested negative by HIV-1 DNA PCR at birth. Clinical notes confirm that his mother was randomized to the breastfeeding arm in the PMTCT Mashi study^[18]^ in the early 2000s. The HIV-1 DNA PCR became positive at 1 month and 2 months after birth suggesting that the transmission of HIV to the child occurred either through intrapartum or early breastfeeding. While on ART and virally suppressed for 14 years, the participant was tested for HIV at a local testing center on his own suspicion that the initial HIV tests were incorrect. However, in the vast majority of cases where virologic confirmation of HIV is made in an infant or child, viral rebound does occur when ART is terminated, even if ART was administered early during HIV infection and participants display very small viral reservoirs.^[4,23,24]^ With most perinatally-infected children now receiving early and long-term treatment, the numbers of cases similar to the one described in this report will likely increase, and caution should be exercised when interpreting “negative” diagnostic results.

The negative HIV-1 rapid test and qualitative HIV-1 DNA PCR results and undetectable viral load should not have been surprising for this teenager. Similar cases of the loss of diagnostic markers have been reported elsewhere in perinatally HIV infected children who started early ART.^[25,26]^ Long-term viral suppression can lead to the absence of circulating HIV-1 RNA and disappearance of laboratory markers, but stable reservoirs of latent virus persist in CD4+ T cells^[24,25]^ and reactivation of these cells can lead to resurgence of the infection when ART is withdrawn. One case of post-ART remission (<20 HIV-1 RNA copies/mL) over 8 years was reported in 2017 in a South African 9-year-old, whose treatment was interrupted after 40 weeks in a clinical trial.^[27]^ Another case of post-treatment control was reported in France of an 18-year-old who started ART at birth and was stopped by her parents at 6 years.^[28]^ These case studies highlight the potential diagnostic complications of the loss of detectable laboratory markers after long-term ART.^[24,29]^ These cases are exceptions and may lead providers/patients to believe that they are cured, but similar to the “Mississippi baby,” we cannot predict if and when the latent virus will rebound. Importantly, for both the Mississippi baby and also for an Early Infant Treatment Cohort followed in Botswana, quantitative HIV DNA PCR positivity from peripheral blood mononuclear cells was detected at about 2 years of age, even when standard qualitative HIV DNA PCR assays were negative.^[20,24]^

Studies have shown that very early ART initiation, such as during acute infection or immediately after birth leads to drastic reductions in DNA set-points, viral reservoirs and has led to longer duration of remission; however, ART interruption almost always lead to viral rebound.^[23,30–32]^ These findings have been similar across HIV subtypes. Our case started ART at 9 months of life, and was virally suppressed for more than 12 years. Although we did not have access to adherence data, the viral load kinetics suggested very good adherence. Other studies have demonstrated even longer duration to rebound.^[23]^ In the case of “Mississipi baby,” viral rebound was observed after 2 years of terminating ART^[33]^ and up to more than 12 years in the French ANRS EPF-CO10 pediatric cohort.^[28]^ Henrich et al observed HIV relapse in individuals initiating extremely early ART, during Fiebig stage 1 (detectable plasma RNA and antibody negative), despite loss of detectable HIV in blood and tissues using molecular and culture-based detection methods. Therefore, there is an urgent need for better HIV reservoir assays performed on standard specimens to distinguish potential or true remissions from latent infections that will ultimately rebound. In our case study, it possible that HIV could be detectable in other compartments other than peripheral blood or with ultrasensitive assays. Undetectable DNA by standard commercial molecular assays; therefore, may not be enough rule out HIV infection. A major focus of the HIV cure studies is to identify potential markers predictive of viral rebound and the development of ultra-sensitive viral reservoir assays such as single copy assay,^[34]^ total DNA quantification, viral reactivation, and growth.^[35–37]^

The case described is the first report in Botswana in which termination of ART following negative HIV tests during long-term ART led to viral rebound. This case provides an important lesson for proper care and management of patients on ART. Improper ART termination in the reported case highlights the necessity for developing sensitive assays able to detect and assess the proviral reservoir. The reported case shows that negative diagnostic results from rapid and DNA PCR tests in individuals on long-term ART could be misleading. Health care providers dealing with individuals on long-term ART should interpret these results cautiously. In the event that a decision is made that it is in the patient's best interest to terminate ART, laboratory monitoring must be continued long-term, to assess possible viral rebound.

## 4 Conclusions

Although long-term ART is associated with reduced size of the proviral reservoir, it may also lead to the inability to detect HIV antibodies and DNA using standard methods. Evaluation for possible functional cure (or for false-positive initial testing) by termination of ART, especially without a highly sensitive quantification of proviral reservoir, may be misleading. It is advisable that standard HIV-1 DNA PCR negative tests in individuals on long-term ART are interpreted with caution, and treatment interruption should be avoided.

## 5 Ethical considerations

The study was approved by the Office of Human Research Administration (OHRA) of the Harvard T.H. Chan School of Public Health and by the Health Research and Development Committee of the Botswana Ministry of Health and Wellness. The parent and participant signed written informed consent and assent, respectively, which includes publication of results where de-identified and de-linked codes will be used.

## Author contributions

**Conceptualization:** Catherine Kegakilwe Koofhethile, Sikhulile Moyo.

**Formal analysis:** Catherine Kegakilwe Koofhethile, Sikhulile Moyo.

**Investigation:** Catherine Kegakilwe Koofhethile, Sikhulile Moyo, Kenanao P. Kotokwe, Patrick Mokgethi, Lorato Muchoba, Selebogo Mokgweetsi, Tendani Gaolathe, Joseph Makhema, Roger Shapiro, Shahin Lockman, Phyllis Kanki, M. Essex, Simani Gaseitsiwe, Tulio de Oliveira, Vladimir Novitsky.

**Methodology:** Catherine Kegakilwe Koofhethile, Sikhulile Moyo.

**Supervision:** Roger Shapiro, Phyllis Kanki, M. Essex, Vladimir Novitsky.

**Writing – original draft:** Catherine Kegakilwe Koofhethile, Sikhulile Moyo.

**Writing – review and editing:** Catherine Kegakilwe Koofhethile, Sikhulile Moyo, Roger Shapiro, Phyllis Kanki, M. Essex, Simani Gaseitsiwe, Vladimir Novitsky.

Catherine Kegakilwe Koofhethile orcid: 0000-0001-5475-413X.

## References

[1] Finzi D Hermankova M Pierson T Identification of a reservoir for HIV-1 in patients on highly active antiretroviral therapy. Science 1997;278:1295–300. 9360927 10.1126/science.278.5341.1295

[2] Chun TW Stuyver L Mizell SB Presence of an inducible HIV-1 latent reservoir during highly active antiretroviral therapy. Proc Natl Acad Sci U S A 1997;94:13193–7. 9371822 10.1073/pnas.94.24.13193 PMC24285

[3] Chomont N El-Far M Ancuta P HIV reservoir size and persistence are driven by T cell survival and homeostatic proliferation. Nat Med 2009;15:893–900. 19543283 10.1038/nm.1972 PMC2859814

[4] Colby DJ Trautmann L Pinyakorn S Rapid HIV RNA rebound after antiretroviral treatment interruption in persons durably suppressed in Fiebig i acute HIV infection brief-communication. Nat Med 2018;24:923–6. 29892063 10.1038/s41591-018-0026-6 PMC6092240

[5] Chun TW Justement JS Murray D Rebound of plasma viremia following cessation of antiretroviral therapy despite profoundly low levels of HIV reservoir: implications for eradication. AIDS 2010;24:2803–8. 20962613 10.1097/QAD.0b013e328340a239 PMC3154092

[6] Harrigan PR Whaley M Montaner JSG Rate of HIV-1 RNA rebound upon stopping antiretroviral therapy. AIDS 1999;13:F59–62. 10371167 10.1097/00002030-199905280-00001

[7] Steingrover R Pogány K Fernandez Garcia E HIV-1 viral rebound dynamics after a single treatment interruption depends on time of initiation of highly active antiretroviral therapy. AIDS 2008;22:1583–8. 18670217 10.1097/QAD.0b013e328305bd77

[8] Hill AL Rosenbloom DI Goldstein E Real-time predictions of reservoir size and rebound time during antiretroviral therapy interruption trials for HIV. PLoS Pathog 2016;12:e1005535. 27119536 10.1371/journal.ppat.1005535 PMC4847932

[9] Li JZ Etemad B Ahmed H The size of the expressed HIV reservoir predicts timing of viral rebound after treatment interruption. AIDS 2016;30:343–53. 26588174 10.1097/QAD.0000000000000953 PMC4840470

[10] World Health Organization, World Health Organization. Progress Report 2016, Prevent HIV, Test and Treat All. 2016.

[11] Guay LA Musoke P Fleming T Intrapartum and neonatal single-dose nevirapine compared with zidovudine for prevention of mother-to-child transmission of HIV-1 in Kampala, Uganda: HIVNET 012 randomised trial. Lancet 1999;354:795–802. 10485720 10.1016/S0140-6736(99)80008-7

[12] Lange JMA Efficacy of three short-course regimens of zidovudine and lamivudine in preventing early and late transmission of HIV-1 from mother to child in Tanzania, South Africa, and Uganda (Petra study): a randomised, double-blind, placebo-controlled trial. Lancet 2002;359:1178–86. 11955535 10.1016/S0140-6736(02)08214-4

[13] Moodley D Moodley J Coovadia H A multicenter randomized controlled trial of nevirapine versus a combination of zidovudine and lamivudine to reduce intrapartum and early postpartum mother-to-child transmission of human immunodeficiency virus type 1. J Infect Dis 2003;187:725–35. 12599045 10.1086/367898

[14] Lallemant M Jourdain G Le Coeur S A trial of shortened zidovudine regimens to prevent mother-to-child transmission of human immunodeficiency virus type 1. N Engl J Med 2000;343:982–91. 11018164 10.1056/NEJM200010053431401

[15] Shaffer N Chuachoowong R Mock PA Short-course zidovudine for perinatal HIV-1 transmission in Bangkok, Thailand: a randomised controlled trial. Lancet 1999;353:773–80. 10459957 10.1016/s0140-6736(98)10411-7

[16] Wiktor SZ Ekpini E Karon JM Short-course oral zidovudine for prevention of mother-to-child transmission of HIV-1 in Abidjan, Cote d’Ivoire: a randomised trial. Lancet 1999;353:781–5. 10459958 10.1016/S0140-6736(98)10412-9

[17] Connor EM Sperling RS Gelber R Reduction of maternal-infant transmission of human immunodeficiency virus type 1 with zidovudine treatment. Pediatric AIDS clinical trials group protocol 076 study group. N Engl J Med 1994;331:1173–80. 7935654 10.1056/NEJM199411033311801

[18] Shapiro RL Thior I Gilbert PB Maternal single-dose nevirapine versus placebo as part of an antiretroviral strategy to prevent mother-to-child HIV transmission in Botswana. AIDS 2006;20:1281–8. 16816557 10.1097/01.aids.0000232236.26630.35

[19] Thior I Lockman S Smeaton LM Breastfeeding plus infant zidovudine prophylaxis for 6 months vs formula feeding plus infant zidovudine for 1 month to reduce mother-to-child HIV transmission in Botswana - a randomized trial: the Mashi study. J Am Med Assoc 2006;296:794–805. 10.1001/jama.296.7.794 16905785

[20] Shapiro RL Lichterfeld M Hughes MD Low HIV reservoir at 84 weeks in very early treated HIV-infected children in Botswana. 25th Conference on Retroviruses and Opportunistic Infections (CROI 2018), Boston 2018;abstract 136.

[21] WHO. Global guidance on Criteria and processes for validation: Elimination of Mother-to-child Transmission of HIV and Syphilis. Second edition 2017. Cancer Detection and Prevention (2017). Available at: 10.1016/S0020-7292(12)61770-6

[22] Cotton MF Rabie H Impact of earlier combination antiretroviral therapy on outcomes in children. Curr Opin HIV AIDS 2015;10:12–7. 25389804 10.1097/COH.0000000000000117 PMC4429285

[23] Henrich TJ Hatano H Bacon O HIV-1 persistence following extremely early initiation of antiretroviral therapy (ART) during acute HIV-1 infection: an observational study. PLoS Med 2017;14:e1002417. 29112956 10.1371/journal.pmed.1002417 PMC5675377

[24] Persaud D Gay H Ziemniak C Absence of detectable HIV-1 viremia after treatment cessation in an infant. N Engl J Med 2013;369:1828–35. 24152233 10.1056/NEJMoa1302976 PMC3954754

[25] Luzuriaga K McManus M Catalina M Early therapy of vertical human immunodeficiency virus type 1 (HIV-1) infection: control of viral replication and absence of persistent HIV-1-specific immune responses. J Virol 2000;74:6984–91. 10888637 10.1128/jvi.74.15.6984-6991.2000 PMC112215

[26] Merchant M Wright M Kabat W Long-term highly suppressed HIV-infected children and adolescents with negative rapid HIV tests due to significant antibody loss. J Clin Virol 2014;59:172–6. 24440176 10.1016/j.jcv.2013.11.012

[27] Violari A Cotton M Kuhn L Viral and host characteristics of a child with perinatal HIV-1 following a prolonged period after ART cessation in the CHER trial. in International AIDS Society, 2017 Available at: 10.1016/S0140-6736(13)61409-9

[28] Frange P Faye A Avettand-Fenoël V HIV-1 virological remission lasting more than 12 years after interruption of early antiretroviral therapy in a perinatally infected teenager enrolled in the French ANRS EPF-CO10 paediatric cohort: a case report. Lancet HIV 2016;3:e49–54. 26762993 10.1016/S2352-3018(15)00232-5

[29] Burgard M Blanche S Jasseron C Performance of HIV-1 DNA or HIV-1 RNA tests for early diagnosis of perinatal HIV-1 infection during anti-retroviral prophylaxis. J Pediatr 2012;160:60-6.e1. 21868029 10.1016/j.jpeds.2011.06.053

[30] Rouzioux C Hocqueloux L Sáez-Cirion A Posttreatment controllers: what do they tell us? Curr Opin HIV AIDS 2015;10:29–34. 25402707 10.1097/COH.0000000000000123

[31] Sáez-Cirión A Bacchus C Hocqueloux L Post-treatment HIV-1 controllers with a long-term virological remission after the interruption of early initiated antiretroviral therapy ANRS VISCONTI study. PLoS Pathog 2013;9:e1003211. 23516360 10.1371/journal.ppat.1003211 PMC3597518

[32] Ananworanich J Chomont N Ann Eller L HIV DNA set point is rapidly established in acute HIV infection and dramatically reduced by early ART. EBioMedicine 2016;11:68–72. 27460436 10.1016/j.ebiom.2016.07.024 PMC5049918

[33] Luzuriaga K Gay H Ziemniak C Viremic relapse after HIV-1 remission in a perinatally infected child. N Engl J Med 2014;372:786–8. 10.1056/NEJMc1413931 PMC4440331 25693029

[34] Kibirige C The use of ultra-sensitive molecular assays in HIV cure-related research. J AIDS Clin Res 2013;Suppl 6:002. 10.4172/2155-6113.S6-002 PMC4198944 25328815

[35] Wang XQ Palmer S Single-molecule techniques to quantify and genetically characterise persistent HIV. Retrovirology 2018;15:3. 29316955 10.1186/s12977-017-0386-x PMC5761141

[36] Hong F Age E Cillo AR Novel assays for measurement of total cell-associated HIV-1 DNA and RNA. J Clin Microbiol 2016;54:902–11. 26763968 10.1128/JCM.02904-15 PMC4809955

[37] Tosiano MA Jacobs JL Shutt KA A simpler and more sensitive single-copy HIV-1 RNA assay for quantification of persistent HIV-1 viremia in individuals on suppressive antiretroviral therapy. J Clin Microbiol 2019;57:e01714–8. 30626659 10.1128/JCM.01714-18 PMC6425167

